# Indochoroiditis—Periodic Ophthalmia in Horses

**Published:** 1883-07

**Authors:** William Oliver Moore


Yours truly,

EHRICK PARMLY, M.D.

### INDOCHOROIDITIS—PERIODIC OPHTHALMIA IN HORSES.

*To the Editor of the Journal of Comparative Medicine and Surgery:*

DEAR SIR:—In your valuable JOURNAL of April, 1883, in the editorial article you speak of Messrs. Hocquard and Bernard's recent investigations on this subject. Since then I have seen their paper on this subject, and I fail to find anything new or startling. They simply arrive at the conclusion that was promulgated in this JOURNAL by me in April, 1881, two years ago. Since that paper was written I have further investigated the disease, and I have not as yet seen anything that would go to show the parasitic origin claimed by Koch, Bertin and others. That organisms may be found in these diseased eyes I do not doubt; but were they conveyed there by instrument during the examination of the eye, or did they exist there before? I think our German theorists are carrying the matter too far. Are the organisms found in the healthy spermatic fluid evidences of disease? That the disease is an indo-choroiditis, no one can doubt, who examines the diseased eye with the ophthalmoscope. I am sorry that our transatlantic friends do not read your able and interesting JOURNAL, for if they did they might keep better posted on current events.

Yours truly,

WILLIAM OLIVER MOORE, M.D.

